# Nanoparticle-Based Immunoengineered Approaches for Combating HIV

**DOI:** 10.3389/fimmu.2020.00789

**Published:** 2020-04-28

**Authors:** Allan Bowen, Elizabeth E. Sweeney, Rohan Fernandes

**Affiliations:** ^1^The George Washington Cancer Center, The George Washington University, Washington, DC, United States; ^2^Department of Medicine, The George Washington University, Washington, DC, United States

**Keywords:** HIV cure strategies, immunoengineering, nanoparticles, immune activation, HAART (highly active antiretroviral therapy), combination therapy for HIV, latency reversing agents

## Abstract

Highly active antiretroviral therapy (HAART) serves as an effective strategy to combat HIV infections by suppressing viral replication in patients with HIV/AIDS. However, HAART does not provide HIV/AIDS patients with a sterilizing or functional cure, and introduces several deleterious comorbidities. Moreover, the virus is able to persist within latent reservoirs, both undetected by the immune system and unaffected by HAART, increasing the risk of a viral rebound. The field of immunoengineering, which utilizes varied bioengineering approaches to interact with the immune system and potentiate its therapeutic effects against HIV, is being increasingly investigated in HIV cure research. In particular, nanoparticle-based immunoengineered approaches are especially attractive because they offer advantages including the improved delivery and functionality of classical HIV drugs such as antiretrovirals and experimental drugs such as latency-reversing agents (LRAs), among others. Here, we present and discuss the current state of the field in nanoparticle-based immunoengineering approaches for an HIV cure. Specifically, we discuss nanoparticle-based methods for improving HAART as well as latency reversal, developing vaccines, targeting viral fusion, enhancing gene editing approaches, improving adoptively transferred immune-cell mediated reservoir clearance, and other therapeutic and prevention approaches. Although nanoparticle-based immunoengineered approaches are currently at the stage of preclinical testing, the promising findings obtained in these studies demonstrate the potential of this emerging field for developing an HIV cure.

## Introduction

Approximately 37 million people worldwide are living with HIV for which there is no practical cure (1). There are two main types of the virus: HIV-1 and HIV-2. HIV-1, which is the focus of cure strategies discussed in this paper, is more prevalent and pathogenic, and primarily infects CD4^+^ T helper cells (2). Other cell populations susceptible to HIV-1 include dendritic cells, macrophages, microglia, and astrocytes although the mechanisms of infection for these cell types are not yet fully understood (3–8). HAART is an effective treatment regimen for HIV-1 (9), however, it allows the virus to remain viable and does not provide a sterilizing or functional cure in patients. Further HAART causes several deleterious comorbidities (10, 11). Since HAART targets the HIV-1 replication cycle, HIV-1 evades targeting by undergoing latency (12, 13). Latent HIV-1 reservoirs are also able to go undetected by the immune system, which increases the risk of a viral rebound. Another confounding factor is the lack of unique surface markers on latently infected cells, which has hindered the development of strategies to generate total viral clearance or permanent latency (14–16). This challenge is supported by the fact that there have been only two well-documented cases wherein patients have experienced total HIV-1 viral clearance. This suggests that it is extremely rare for individuals to adequately control HIV-1 without a sustained antiretroviral treatment regimen (17, 18). Hence, there is an urgent need for novel HIV cure strategies.

**Figure 1:**
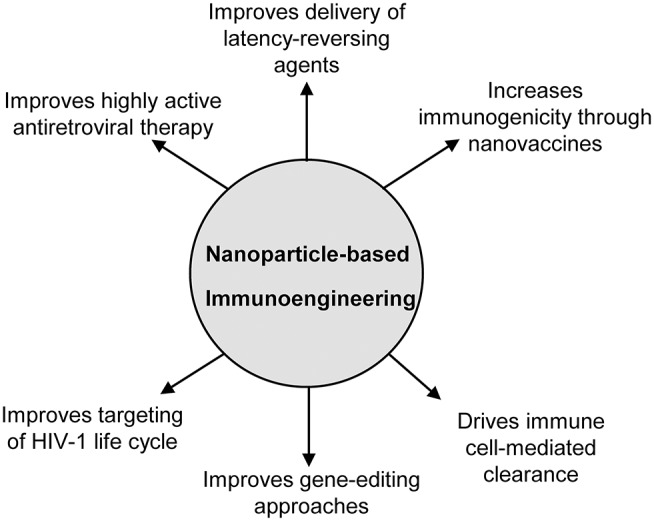
Overview of the applications of nanoparticle-based immunoengineering approaches for combating HIV-1.

The field of immunoengineering encompasses a broad variety of bioengineering approaches and technologies to manipulate the immune system. A notable component of these approaches involves engineered biomaterials including nanoparticles, polymeric scaffolds, and hydrogels to engage the immune system to fight disease, and represents an attractive strategy for developing a cure for HIV-1 (19–21). While numerous nanoparticle-based immunoengineered approaches have been successfully applied in the field of cancer immunotherapy (22–26), fewer studies have utilized these promising benefits for an HIV cure. The goal of this mini-review is to familiarize the reader with the field of nanoparticle-based immunoengineering approaches for an HIV cure. In particular, we focus on the use of nanoparticles to enhance HAART, latency reversal, vaccination strategies, gene editing, cell therapies, among others (Figure 1). We highlight both immune-mediated strategies (e.g., nanoparticles in conjunction with adoptive cell transfer) and those targeting endogenous immune cells involved in HIV (e.g., nanovaccines). For a comprehensive discussion on the use of nanoparticles for treating HIV/AIDS in the context of nanoparticles classes, formulation, and their use in drug delivery, we direct the readers to several published reviews in the literature (27–29).

## Nanoparticles Exhibit Beneficial Properties for Immunoengineering Cures for HIV-1

Nanoparticle sizes range from ~1 to 100 nm (30). On account of their sizes, nanoparticle-based therapies can easily be administered by varied techniques (i.e., intravenously, subcutaneously, intraperitoneally) and penetrate body barriers (31). Related to their sizes and pertinent to HIV cure strategies, nanoparticles accumulate in lymphoid tissue and lymphatic organs (32), the sites of anatomical HIV reservoirs (33, 34) when parenterally injected (especially when injected intradermally, subcutaneously, and/or intramuscularly). Additionally, because nanoparticles have a large surface area-to-volume ratio, diverse molecules such as drug payloads, immunological adjuvants, and targeting ligands can be bioconjugated to their surface (35), which can then be trafficked to sites of latent HIV reservoirs. Nanoparticles can also be synthesized in the form of depots/reservoirs that encapsulate and release therapeutic drugs or immunomodulatory agents (36), allowing for their improved bioavailability and sustained release kinetics in an HIV cure setting. In the following sections, we review various examples of nanoparticles used for immunoengineering HIV cures.

### Nanoparticles for Improving HAART

HAART can successfully inactivate HIV-1, however the virus is able to persist within latent lymphoid, gut, and CNS reservoirs (5–8). This requires patients to remain adherent to the HAART regimen for the duration of their lifetime to prevent viral rebound (9). Since nanoparticles facilitate the sustained release of drugs, a group of researchers have developed a long-acting slow-effective release antiretroviral therapy (termed “LASER ART”), which utilizes nanoparticles for controlled release of HAART agents, thereby improving regimen adherence (37, 38). In a recent study, the same group of researchers demonstrated that a nanocrystallized product of lamivudine, a nucleoside reverse transcriptase inhibitor (NM23TC), maintained antiretroviral activity in HIV-1-infected monocyte-derived macrophages after viral challenge for up to 30 days. NM23TC was taken up by HIV-1-infected monocyte-derived macrophages and remained in high prodrug concentration in whole blood for 30 days after a single dose. In addition, at day 28, M23TC, a metabolized version of lamivudine, was detected at high levels in the liver, lymph nodes, and spleen (39), suggesting that this nanoplatform may improve delivery of the drug to HIV-1 niches. Other groups have also developed nanoparticles for improving HAART (40–43) (Table 1).

**Table 1:** Nanoparticle-based immunoengineering approaches for HIV/AIDS.

**Nanoparticle class**	**Key findings**	**References**
**NANOPARTICLES FOR IMPROVING HAART**		
Poloxamer-based nanoparticles	LASER ART (also known as nanoART) nanoparticles for controlled release of HAART agents	(37–40)
Lipid nanoparticle	Lipid-drug nanoparticles exhibited 5-fold increased bioavailability of HAART drugs in lymph nodes, and markedly increased sustained release over the course of 7 days	(41)
PLGA/pluronic nanoparticle	Nanoformulation of HAART drugs demonstrate greater bioavailability in plasma and absorption in various tissues over the course of 14 days	(42)
Lactoferrin nanoparticle	First-line nanoformulated HAART drugs exhibited 4-fold increase in bioavailability and an increase in anti-HIV activity compared to soluble agents	(43)
**NANOPARTICLES FOR IMPROVING LATENCY REVERSAL**		
Lipid nanoparticle	LRA and protease inhibitor encapsulated within nanoparticle reversed latency and prevented HIV-1 viral spread	(44)
Lipid-coated PLGA nanoparticle	Co-administration of LRAs-individually encapsulated within nanoparticles exhibited synergistic induction of HIV-1 mRNA levels at low cytotoxicity	(45)
Iron oxide nanoparticle	LRA and vorinostat-loaded nanoparticle penetrated BBB, reversed HIV-1 latency and exhibited antiviral efficacy in astrocytes.	(46)
PLG/PEG nanoparticle	LRA and protease inhibitor encapsulated within PLGA-PEG nanoparticles reversed latency and inhibited viral spread	(47)
**NANOVACCINES**		
PLGA nanoparticle	Encapsulating TLR-agonist improved HIV-1 vaccine immunogenicity and decrease required dose for immunogenic effect	(48)
Lumazine synthase- and ferritin-based nanoparticles	Antigens encapsulated within nanoparticles were trafficked within germinal centers promoting a potent immunogenic response	(49)
Polyethylenimine mannose/DNA/glucose nanoparticle	DermaVir nanoformulation delivered HIV-1 antigen to Langerhans cells, which matured into Dendritic cells, mounting an immune response	(50)
eOD-GT8 nanoparticle	Engineered outer domain (eOD)-60 mer nanoparticle exhibited sufficient precursor naïve B cell binding for bnAb production	(51)
Liposome nanoparticle	Clade C-derived trimers decorated on liposomal surface induced enhanced germinal center and bnAb responses compared to soluble trimers	(52)
Ferritin nanoparticle	Consensus-derived Env trimers conjugated to nanoparticles induced greater bnAb targeting of apex trimers of *in vivo* models	(53)
Protein nanoparticle	Nanomaterial presenting SOSIP trimer increased B-cell activation and induced greater bnAb titers against Tier-1A viral strains	(54)
Liposome nanoparticle	Vaccination with liposomes formulated with HIV envelope protein elicits bnAb targeting and neutralization	(55)
Ferritin nanoparticle	HIV antigens are presented on nanoparticles in native trimeric structure as a tool for vaccine development	(56)
**NANOPARTICLES TARGETING HIV VIRAL FUSION TO IMMUNE CELLS**		
Silver nanoparticle	Silver nanoparticles exert anti-HIV activity through gp120 binding in various viral strains	(57)
Poly (acrylate)-based nanoparticle	Hydrophobic nanoparticle impedes amyloid fiber structure, thereby disrupting HIV-1 trafficking to its target cell	(58)
PLGA nanoparticle	Nanoparticles coated with a T-cell membrane were able to serve as a “decoy” for HIV-1 binding, resulting in viral suppression	(59)
Extracellular vesicles	Extracellular vesicles (EVs) released by *Lactobacillus* inhibited HIV-1 viral attachment and entry to target cells	(60)
Extracellular vesicles	EVs isolated from semen inhibited HIV-1 regardless of donor infection status; EVs from ART-treated subjects inhibited HIV-1 *in vivo*	(61)
**NANOPARTICLES TO ENHANCE GENE EDITING APPROACHES**		
Gold nanoparticles	Au-nanoparticles can mediate CRISPR-Cas9 components to target cells with higher efficiency and lower cytotoxicity	(62)
Poloxamer-based nanoparticles	LASER ART combined with CRISPR/Cas9 eliminated HIV-1 in a small subset of mice	(63)
**NANOPARTICLES TO ENHANCE CLEARANCE BY ADOPTIVELY**		
**TRANSFERRED IMMUNE CELLS**		
PLGA nanoparticles	Nanoparticles encapsulating neutralizing antibody and LRA improved NK cell effector function toward J-Lat cells compared to free agents	(64)
Lipid nanoparticle	IL-15-loaded nanocapsules conjugated to HIV-1-specific CTLs improved elimination of infected cells	(65)
**OTHER THERAPIES**		
PEG-*b*-PR co-polymer nanoparticles	STING agonist nanoformulation reversed HIV-1 immune evasion mechanism	(66)
Quantum dots	Graphene quantum dots mediated HIV-1 viral suppression	(67)
Nanodiamonds	Efavirenz-nanodiamond conjugation improved bioavailability and blood brain barrier penetration	(68)
Gold nanoparticles	Gold conjugated with HIV integrase inhibitors could penetrate the BBB and exert antiviral efficacy in targeted HIV-1-infected microglial cells	(69)
PLGA nanoparticles	FTC-loaded nanoparticles exhibited greater bioavailability and lower IC_50_ compared to soluble agents	(70)
PLGA nanoparticles	TDF-loaded nanoparticles in thermosensitive gel conferred 100% protection from HIV-1 strains within 24 h time period and had no detectable viral levels in plasma throughout 4 weeks period	(71)
Cellulose acetate phthalate nanoparticles	DTG-loaded nanoparticles in thermosensitive gel were uptaken into vaginal epithelial cells with low cytotoxicity	(72)

### Nanoparticles for Improving Latency Reversal

Latency-reversing agents (LRAs) are able to reactivate viral replication. LRAs are used in “shock and kill” treatment approaches, wherein the LRA-elicited viral replication is coupled to the actions of HIV-1 cell-specific cytotoxic agents or immune-mediated clearance (73). Several classes of molecules and macromolecules have been used as LRAs including protein kinase C (PKC) agonists, histone deacetylase inhibitors, and cytokines, and their mechanisms of latency reversal are well-described. For example, PKC agonists function via the NF-KB pathway. Activated PKC isoforms downregulate the inhibitor IKB, thereby releasing the transcription factor NF-KB, which translocates into the nucleus, and binds to the HIV-1 proviral long terminal repeats, thereby mediating viral transcription (74). Similar to HAART, nanoparticles have been utilized to improve the delivery of LRAs (Table 1). In one study, Kovochich et al. encapsulated bryostatin, a potent PKC agonist (75) and nelfinavir, an HIV-1 protease inhibitor, into nanoparticles. Their nanoplatform targeted CD4^+^ cells in a peripheral blood mononuclear cells (PBMC) culture, activated latent virus, and inhibited viral spread (76). In a more recent study, Cao et al. synthesized hybrid lipid-coated PLGA nanocarriers that incorporated diverse LRAs. These lipid-coated nanoparticles could selectively activate CD4^+^ T cells in nonhuman primate PBMCs as well as in murine lymph nodes with substantially reduced toxicity (44). Despite the fact that it is currently impossible to identify and target every HIV-1-infected cell in the latent reservoir (45), the ability of nanoparticles and nanocarriers to traffic and deliver LRAs to sites of latent HIV reservoirs can maximize their therapeutic benefit, and serve as an important component of successful shock and kill cure regimens. Other examples of nanoparticles for improving latency reversal appear in Table 1 (46, 47).

### Nanovaccines

Traditional vaccines for HIV-1 have been difficult to develop, and clinical trials using HIV-1 vaccines have demonstrated poor efficacy (77, 78). Vaccines fail for several reasons including poor delivery to dendritic cells, reversion of a live attenuated virus to its virulent form, or if the vaccine is too weak to facilitate an immune response (68). Consequently, an ideal vaccine should be clinically safe, stable, and capable of inducing a potent immune response (79). Nanoparticles have been shown to overcome these limitations (80) by protecting antigens from proteolytic enzymes, promoting antigen uptake and processing by antigen-presenting cells (APCs), in addition to being biocompatible and biodegradable (81). Several groups have leveraged favorable properties of nanoparticles to develop nanovaccines for HIV-1 (Table 1).

One effective strategy is utilizing nanovaccines to activate dendritic cells (DCs), which in turn cause T cell activation (82). To this end, Rostami et al. decorated antigens onto the surface of nanoparticles to facilitate greater interaction with the APCs due to the high surface area to volume ratio of the nanoparticles (48). Specifically, a flagellin molecule sequence derived from *Pseudomonas aeruginosa* (FLiC), a toll-like receptor 5 agonist, was conjugated to an HIV-1 p24-NeF peptide, and encapsulated within PLGA nanoparticles. The FLiC-p24-NeF-encapsulated nanoparticle elicited higher levels of lymphocyte proliferation and cytotoxic T cell activity compared to controls (48), suggesting its potential use in an HIV-1 vaccination strategy. In a more recently study by Tokatlian et al., nanoparticles encapsulating HIV-1 antigens were observe to localize to the lymph nodes more than corresponding soluble antigen counterparts, and remained localized there for up to 4 weeks (49). In another study, Lori et al. showed that their nanoplatform “DermaVir” could administer HIV-1 antigens to Langerhans cells, which resulted in a potent immunogenic response (50). DermaVir is currently undergoing a phase 3 clinical trial evaluation based on excellent responses observed in Phase I/II clinical trials (83). Together, these studies along with others summarized in Table 1 (51–56), clearly suggest the importance of nanovaccines for treating HIV-1.

### Nanoparticles Targeting HIV Viral Fusion to Immune Cells

Targeting the HIV replication cycle by inhibiting the ability of HIV-1 to fuse and/or enter a target cell has been the focus of several published studies (Table 1). Fusion or entry inhibition leads to inhibition of viral activity and viral cytotoxicity. In one approach, Lara et al. showed that silver nanoparticles are antiviral and prophylactic against HIV-1 fusion to target cells (57). Silver nanoparticles exert anti-HIV activity at an early stage of viral replication, likely as a virucidal agent or as an inhibitor of viral entry. Silver nanoparticles bind to gp120 in a manner that prevents CD4-dependent virion binding, fusion, and infectivity, acting as an effective virucidal agent against cell-free and cell-associated virus. Further, silver nanoparticles inhibit post-entry stages of the HIV-1 life cycle (57).

Another approach utilized semen-derived enhancer of viral infection (SEVI), which is a type of amyloid fibril present in human semen that enhances HIV-1 infection of target cells by capturing HIV-1 virions, resulting in increased viral fusion (84). SEVI serves as a mediator for HIV-1 viral attachment due to its highly cationic nature (84, 85). In their study, Sheik et al., synthesized a hydrophobic polymeric nanoparticle to reduce SEVI fibril-mediated infection (58). The hydrophobicity of the nanoparticle interferes with Aβ amyloid structure, forming amorphous aggregates, thereby disrupting the amyloid HIV-1 trafficking protein to target cells (86–88). Thus, the hydrophobic nanoparticles were able to reduce HIV-1 virion binding affinity toward their target cells (58).

Biomimicry approaches, such as plasma membrane-coated nanoparticles, represent a unique strategy to target a variety of human pathologies (89). A pivotal study showed the efficacy of coating a nanoparticle with a cell membrane to imitate and model endogenous cell activity. HIV-1 infection begins when an exposed HIV-1 surface protein, gp120, interacts with CD4 receptor and chemokine receptor type 5 (CCR5) co-receptor on target cells (90). Wei et al. coated polymeric nanoparticles with a CD4^+^ T cell membrane, causing the modified membrane-coated nanoparticle to preferentially interact with HIV-1. This preferential binding ultimately neutralized HIV-1 viral activity in PBMCs *in vitro* (59), illustrating the potential of biomimicking nanoparticle approaches to reduce HIV-1 viral spread by blocking viral fusion to T cells.

Unlike synthetic nanoparticles, extracellular vesicles (EVs) are naturally occurring nanoscale structures that carry cargo (e.g., proteins, lipids, nucleic acids) and can be released from both healthy and apoptotic cells (91). Recently, Palomino et al. discovered that EVs released by Lactobacillus in the healthy vaginal microbiota prevented HIV-1 attachment to target cells and thereby inhibited HIV-1 infection (60). In a recent study by Welch et al., EVs extracted from semen inhibited HIV-1 *in vitro* regardless of HIV infection status of the donor, while EVs extracted from the blood and semen of ART-treated subjected inhibited HIV-1 *in vivo* (61). These studies suggest a potential avenue for bacterial and/or EV-based treatment strategies in preventing HIV-1 viral spread.

### Nanoparticles to Enhance Gene Editing Approaches

Gene therapy technologies have been explored for HIV-1 cure strategies (Table 1). Clustered regularly interspaced short palindromic repeats and CRISPR-associated protein 9 (CRISPR-Cas9) is a gene editing platform wherein genes can be added, removed, or altered at given genetic loci (92). CRISPR-Cas9 is a faster and more efficient technique than other genetic editing platforms using other viral vectors or the Cre-Lox system, although current CRISPR-Cas9 delivery techniques use electroporation to facilitate DNA entry into living cells, which is difficult to control and can generate cytotoxicity (92, 93). Previously, gene-editing tools have knocked out CCR5 in CD4^+^ T cells to block HIV-1 viral entry (94, 95). However, gold nanoparticles (AuNPs) have a unique ability to safely deliver CRISPR-Cas9 components to their targets (96). AuNPs have large surface area to volume ratios and are biocompatible with low toxicity (5). Shahbazi et al. developed AuNPs with layer-by-layer surface conjugation of CRISPR components (AuNP/CRISPR), targeting two locations within the hematopoietic stem and progenitor cell (HSPC) genome, CCR5 and the gamma-globin gene promoter. Genetic deficiency in CCR5 is linked to HIV-1 resistance through the elimination of viral anchoring and entry through its CCR5 co-receptor (62, 97). AuNP/CRISPR was able to penetrate into CD34^+^ hematopoietic cell line, which is difficult to transfect. At micromolar concentrations, AuNP/CRISPR exhibited an overall low amount of gene editing and homologous directed repair (HDR) at the CCR5 and the gamma-globin promoter locus. This demonstrates that AuNP/CRISPR functioned with low efficacy. However, genetic editing and HDR via AuNP/CRISPR was higher than the electroporation-driven process. This suggests that AuNP/CRISPR could be effective in delivering gene editing for HIV-1 therapy (62).

With the promising innovations of LASER ART and CRISPR-Cas9, Dash et al. combined the two methodologies to evaluate a potentially synergistic functionality. Two of seven HIV-1-infected mice that received LASER ART followed by subsequent AAV_9_-CRISPR-Cas9 treatment targeting a fragment of the HIV-1 genome were cured of viral rebound and experienced a restoration of their CD4^+^ T cells, suggesting HIV-1 regression/elimination (63). Additionally, HIV-1 RNA levels diminished to undetectable levels in the plasma, spleen, liver, gut, and brain in the cured mice. Further, naïve humanized mice that were challenged with adoptively transferred cells isolated from the cured mice showed no detectable HIV-1 viral loads. This study demonstrates the possibility of eliminating HIV-1 in plasma and infectious tissues through this novel combination approach (63).

### Nanoparticles to Enhance Clearance by Adoptively Transferred Immune Cells

Recent studies show promising effects of cell therapies for treating HIV-1 (98–100). Here, autologous or allogenic immune cells are transferred to the patient after *ex vivo* expansion and/or modification to clear HIV-1 infected cells. Nanoparticles may offer the ability to enhance the ability of immune cells to target and kill target cells in the context of HIV-1. In their study, Sweeney et al. generated a PLGA nanoplatform that co-encapsulated an LRA and a target cell-specific antibody to improve NK cell effector function in an *in vitro* cell model of latent HIV-1 (64). The nanoplatform was able to increase NK cell cytotoxicity of the target cells, thereby illustrating an example of nanoparticles enhancing immune cell function in the context of latent HIV-1 (64). In another studies, Jones et al. demonstrated that cytotoxic T lymphocytes (CTLs) were made more potent by conjugating drug-loaded lipid nanoparticles to their surface (65). HIV-1-specific CTLs were able to specifically target HIV-1-infected cells and deliver the nanoparticle-encapsulated payload (65).

### Other Focus Areas

#### Nanoparticles to Boost Innate Immunity

HIV-1, like many other viruses, has evolved mechanisms to evade or disrupt immune surveillance. Therefore, one strategy to eliminate HIV-1 viral load is to reverse immune evasion. HIV-1 typically inhibits the cGAS-STING pathway that normally functions via cGAMP binding to STING on the endoplasmic reticulum resulting in an IFN-1-mediated antiviral response (66, 101, 102). pH-sensitive polymeric nanoparticles were engineered to deliver a STING agonist to counteract HIV-1 immune evasion via the cGAS-STING pathway. These STING agonist-nanoparticles demonstrated potent antiretroviral activity for up to 12 days (66).

#### Nanoparticles to Inhibit HIV-1 Reverse Transcriptase Activity

Quantum dots are biocompatible semiconductor crystal nanoparticles with low toxicity that have been used for biosensing, image contrast, and drug delivery (103, 104). These nanomaterials are attractive due to their intrinsic antiviral activity and thus, their potential as inhibitors of HIV-1. In a proof-of-concept study by Iannazzo et al., a reverse transcriptase inhibitor (RTI; CHI499) was readily conjugated onto the surface of the graphene quantum dots (GQDs) via intrinsic functional groups (67). The conjugated GQD product (GQD-CHI499) achieved remarkable anti-reverse transcriptase and cellular anti-HIV-1 activities compared to the free drug alone. This additive improvement may be the result of the GQDs' intrinsic structure, where the polycarboxylation group could mediate the inhibition of HIV-1 reverse transcriptase through viral fusion (67), suggesting the potential of GQDs in treatment for HIV-1.

#### Nanoparticles to Enhance Blood Brain Barrier Penetration

Aside from persistent HIV-1 in CD4^+^ helper T cells, HIV-1 may also persist in microglial cells, which are the resident macrophages of the CNS (6). These cells may confer HAART resistance, perpetuate HIV-1 infection in peripheral tissues, and are critical in the development of HIV-1 associated neurocognitive diseases (105). The brain poses an anatomical barrier, where there is low drug penetration by virtue of the blood brain barrier (BBB). Therefore, there is a need to develop ways to penetrate the BBB to target persistent HIV-1 in microglial cells (6, 105). Nanodiamonds are ~10 nm diamonds which are known for their inexpensive production, surface modifications, and low cytotoxic profile. In the context of HIV-1, Roy et al. complexed efavirenz (EFV), an effective non-nucleoside RTI, to a nanodiamond to effectively improve the poor bioavailability of EFV (ND-EFV) (68). ND-EFV allowed for sustained release of EFV in a BBB model *in vitro*. In addition, ND-EFV was effective in controlling HIV-1 replication for 7 days, where the EFV alone drug was able to inhibit HIV-1 for 5 days (68). Similarly, gold nanoparticles have also been used entry through the BBB. Garrido et al. showed that gold nanoparticles conjugated with HIV integrase inhibitors could penetrate the BBB with antiviral efficacy, providing another nanoplatform for targeting HIV-1-infected microglial cells (69).

#### Nanoparticles for Prophylactic HIV-1 Prevention

Another application of nanoparticles is in improving pre-exposure prophylaxis (PrEP), which provides a >90% effective approach to prevent HIV-1 infection but requires daily oral administration (106). PrEP comprises tenofovir disoproxil fumarate (TDF) and emtricitabine (FTC), two nucleoside RTIs that have low half-lives and require high dosing, thereby increasing the risk of adverse effects (107). Mandal et al. encapsulated FTC within PLGA nanoparticles (FTC-NPs), and demonstrated improved bioavailability of FTC with significantly lower inhibitory concentration (IC_50_) than free FTC *in vitro* (70). Alternatively, Destache et al. loaded TDF into PLGA nanoparticles (TDF-NPs), and subsequently incorporated them within a thermosensitive vaginal gel (71). Mice challenged with two strains of HIV-1 and treated with the TDF-NP gel were 100% protected against the virus with no detectable viral plasma load (71), suggesting the efficacy of sustained release of TDF by nanoparticles via vaginal administration to prevent HIV-1 infection. In another study, Mandal et al. encapsulated dolutegravir (DTG), an integrase strand transfer inhibitor, within nanoparticles made from cellulose acetate phthalate, a pH-sensitive polymer that intrinsically inhibits HIV-1 entry into its target cells (DTG-CAP-NPs) (72). Similarly to above, DTG-CAP-NPs were incorporated into a thermosensitive vaginal gel. Vaginal epithelial cells were able to take up DTG-CAP-NPs, where they persisted for up to 7 days with low cytotoxicity (72). These studies demonstrate the potential of nanoparticles for use in HIV-1 preventative strategies, including enhancing PrEP.

## Conclusion

Here, we have reviewed the field of nanoparticle-based immunoengineered approaches toward an HIV-1 cure. We highlighted the potential and use of nanoparticles to facilitate and improve the delivery, bioavailability, and/or functionality of HAART, LRAs, vaccines, gene-editing approaches, and other therapeutic or preventative strategies. The innovative advances described herein demonstrate the potential of the field of nanoparticle-based immunoengineering in treating and preventing HIV-1.

## Author Contributions

AB, ES, and RF all participated in the writing and the preparation of the manuscript, and approved it for publication.

## Conflict of Interest

The authors declare that the research was conducted in the absence of any commercial or financial relationships that could be construed as a potential conflict of interest.

## References

[1] Jordan A Bisgrove D Verdin E. HIV reproducibly establishes a latent infection after acute infection of T cells *in vitro*. EMBO J. (2003) 22:1868–77. 10.1093/emboj/cdg188 12682019 PMC154479

[2] Martinez Rivas CJ Tarhini M Badri W Miladi K Greige-Gerges H Nazari QA. Nanoprecipitation process: from encapsulation to drug delivery. Int J Pharm. (2017) 532:66–81. 10.1016/j.ijpharm.2017.08.064 28801107

[3] Fredenberg S Wahlgren M Reslow M Axelsson A. The mechanisms of drug release in poly(lactic-co-glycolic acid)-based drug delivery systems–a review. Int J Pharm. (2011) 415:34–52. 10.1016/j.ijpharm.2011.05.049 21640806

[4] Wiemann B Starnes CO. Coley's toxins, tumor necrosis factor and cancer research: a historical perspective. Pharmacol Ther. (1994) 64:529–64. 10.1016/0163-7258(94)90023-X 7724661

[5] Murphy CJ Gole AM Stone JW Sisco PN Alkilany AM Goldsmith EC. Gold nanoparticles in biology: beyond toxicity to cellular imaging. Acc Chem Res. (2008) 41:1721–30. 10.1021/ar800035u 18712884

[6] Wallet C De Rovere M Van Assche J Daouad F De Wit S Gautier V. Microglial cells: the main HIV-1 reservoir in the brain. Front Cell Infect Microbiol. (2019) 9:362. 10.3389/fcimb.2019.00362 31709195 PMC6821723

[7] Chauveau L Donahue DA Monel B Porrot F Bruel T Richard L. HIV fusion in dendritic cells occurs mainly at the surface and is limited by low CD4 levels. J Virol. (2017) 91:e01248-17. 10.1128/JVI.01248-17 28814521 PMC5640872

[8] Wu L KewalRamani VN. Dendritic-cell interactions with HIV: infection and viral dissemination. Nat Rev Immunol. (2006) 6:859–68. 10.1038/nri1960 17063186 PMC1796806

[9] Chesney M. Adherence to HAART regimens. Aids Patient Care St. (2003) 17:169–77. 10.1089/108729103321619773 12737640

[10] Sension MG. Long-Term suppression of HIV infection: benefits and limitations of current treatment options. J Assoc Nurses AIDS Care. (2007) 18(1 Suppl.):S2–10. 10.1016/j.jana.2006.11.012 17275719

[11] Margolis AM Heverling H Pham PA Stolbach A. A review of the toxicity of HIV medications. J Med Toxicol. (2014) 10:26–39. 10.1007/s13181-013-0325-8 23963694 PMC3951641

[12] Reeves DB Duke ER Wagner TA Palmer SE Spivak AM Schiffer JT. A majority of HIV persistence during antiretroviral therapy is due to infected cell proliferation. Nat Commun. (2018) 9:4811. 10.1038/s41467-018-06843-5 30446650 PMC6240116

[13] Richman DD Margolis DM Delaney M Greene WC Hazuda D Pomerantz RJ. The challenge of finding a cure for HIV infection. Science. (2009) 323:1304–7. 10.1126/science.1165706 19265012

[14] Siliciano RF Greene WC. HIV latency. Cold Spring Harb Perspect Med. (2011) 1:a007096. 10.1101/cshperspect.a007096 22229121 PMC3234450

[15] Williams SA Chen LF Kwon H Ruiz-Jarabo CM Verdin E Greene WC. NF-kappaB p50 promotes HIV latency through HDAC recruitment and repression of transcriptional initiation. EMBO J. (2006) 25:139–49. 10.1038/sj.emboj.7600900 16319923 PMC1356344

[16] Dahabieh MS Battivelli E Verdin E. Understanding HIV latency: the road to an HIV cure. Annu Rev Med. (2015) 66:407–21. 10.1146/annurev-med-092112-152941 25587657 PMC4381961

[17] Gupta RK Abdul-Jawad S McCoy LE Mok HP Peppa D Salgado M. HIV-1 remission following CCR5Delta32/Delta32 haematopoietic stem-cell transplantation. Nature. (2019) 568:244–8. 10.1038/s41586-019-1027-4 30836379 PMC7275870

[18] Hutter G Nowak D Mossner M Ganepola S Mussig A Allers K. Long-term control of HIV by CCR5 Delta32/Delta32 stem-cell transplantation. N Engl J Med. (2009) 360:692–8. 10.1056/NEJMoa0802905 19213682

[19] Goldberg MS. Immunoengineering: how nanotechnology can enhance cancer immunotherapy. Cell. (2015) 161:201–4. 10.1016/j.cell.2015.03.037 25860604

[20] Delcassian D Sattler S Dunlop IE. T cell immunoengineering with advanced biomaterials. Integr Biol (Camb). (2017) 9:211–22. 10.1039/c6ib00233a 28252135 PMC6034443

[21] Xie YQ Wei L Tang L. Immunoengineering with biomaterials for enhanced cancer immunotherapy. Wiley Interdiscip Rev Nanomed Nanobiotechnol. (2018) 10:e1506. 10.1002/wnan.1506 29333729

[22] Wang C Xu L Liang C Xiang J Peng R Liu Z. Immunological responses triggered by photothermal therapy with carbon nanotubes in combination with anti-CTLA-4 therapy to inhibit cancer metastasis. Adv Mater. (2014) 26:8154–62. 10.1002/adma.201402996 25331930

[23] Chen Q Xu L Liang C Wang C Peng R Liu Z. Photothermal therapy with immune-adjuvant nanoparticles together with checkpoint blockade for effective cancer immunotherapy. Nat Commun. (2016) 7:13193. 10.1038/ncomms13193 27767031 PMC5078754

[24] Nam J Son S Ochyl LJ Kuai R Schwendeman A Moon JJ. Chemo-photothermal therapy combination elicits anti-tumor immunity against advanced metastatic cancer. Nat Commun. (2018) 9:1074. 10.1038/s41467-018-03473-9 29540781 PMC5852008

[25] Sweeney EE Cano-Mejia J Fernandes R. Photothermal therapy generates a thermal window of immunogenic cell death in neuroblastoma. Small. (2018) 14:e1800678. 10.1002/smll.201800678 29665282

[26] Cano-Mejia J Bookstaver ML Sweeney EE Jewell CM Fernandes R. Prussian blue nanoparticle-based antigenicity and adjuvanticity trigger robust antitumor immune responses against neuroblastoma. Biomater Sci. (2019) 7:1875–87. 10.1039/C8BM01553H 30789175 PMC6491208

[27] Mamo T Moseman EA Kolishetti N Salvador-Morales C Shi J Kuritzkes DR. Emerging nanotechnology approaches for HIV/AIDS treatment and prevention. Nanomedicine (Lond). (2010) 5:269–85. 10.2217/nnm.10.1 20148638 PMC2861897

[28] Victor OB Nanoparticles and its implications in HIV/AIDS therapy. Curr Drug Discov Technol. (2019) 16:1 10.2174/1570163816666190620111652

[29] Cao S Woodrow KA. Nanotechnology approaches to eradicating HIV reservoirs. Eur J Pharm Biopharm. (2019) 138:48–63. 10.1016/j.ejpb.2018.06.002 29879528 PMC6279622

[30] International Organization for Standardization (ISO) Nanotechnologies — Vocabulary — Part 2: Nano-objects. ISO/TS 80004-2:2015 (2015).

[31] Blanco E Shen H Ferrari M. Principles of nanoparticle design for overcoming biological barriers to drug delivery. Nat Biotechnol. (2015) 33:941–51. 10.1038/nbt.3330 26348965 PMC4978509

[32] Rao DA Forrest ML Alani AW Kwon GS Robinson JR. Biodegradable PLGA based nanoparticles for sustained regional lymphatic drug delivery. J Pharm Sci. (2010) 99:2018–31. 10.1002/jps.21970 19902520 PMC5178132

[33] Vanhamel J Bruggemans A Debyser Z. Establishment of latent HIV-1 reservoirs: what do we really know? J Virus Erad. (2019) 5:3–9. 30800420 10.1016/S2055-6640(20)30275-2 PMC6362902

[34] Pantaleo G Graziosi C Butini L Pizzo PA Schnittman SM Kotler DP. Lymphoid organs function as major reservoirs for human immunodeficiency virus. Proc Natl Acad Sci USA. (1991) 88:9838–42. 10.1073/pnas.88.21.9838 1682922 PMC52816

[35] Sinha R Kim GJ Nie S Shin DM. Nanotechnology in cancer therapeutics: bioconjugated nanoparticles for drug delivery. Mol Cancer Ther. (2006) 5:1909–17. 10.1158/1535-7163.MCT-06-0141 16928810

[36] Reis CP Neufeld RJ Ribeiro AJ Veiga F. Nanoencapsulation I. Methods for preparation of drug-loaded polymeric nanoparticles. Nanomedicine. (2006) 2:8–21. 10.1016/j.nano.2005.12.003 17292111

[37] Edagwa B McMillan J Sillman B Gendelman HE. Long-acting slow effective release antiretroviral therapy. Expert Opin Drug Deliv. (2017) 14:1281–91. 10.1080/17425247.2017.1288212 28128004 PMC5545165

[38] Gnanadhas DP Dash PK Sillman B Bade AN Lin Z Palandri DL. Autophagy facilitates macrophage depots of sustained-release nanoformulated antiretroviral drugs. J Clin Invest. (2017) 127:857–73. 10.1172/JCI90025 28134625 PMC5330738

[39] Smith N Bade AN Soni D Gautam N Alnouti Y Herskovitz J. A long acting nanoformulated lamivudine ProTide. Biomaterials. (2019) 223:119476. 10.1016/j.biomaterials.2019.119476 31525692 PMC6945491

[40] Guo D Zhou T Arainga M Palandri D Gautam N Bronich T. Creation of a long-acting nanoformulated 2',3'-dideoxy-3'-thiacytidine. J Acquir Immune Defic Syndr. (2017) 74:e75–e83. 10.1097/QAI.0000000000001170 27559685 PMC5305294

[41] Freeling JP Koehn J Shu C Sun J Ho RJ. Long-acting three-drug combination anti-HIV nanoparticles enhance drug exposure in primate plasma and cells within lymph nodes and blood. Aids. (2014) 28:2625–7. 10.1097/QAD.0000000000000421 25102089 PMC4376321

[42] Prathipati PK Mandal S Pon G Vivekanandan R Destache CJ. Pharmacokinetic and tissue distribution profile of long acting tenofovir alafenamide and elvitegravir loaded nanoparticles in humanized mice model. Pharm Res. (2017) 34:2749–55. 10.1007/s11095-017-2255-7 28905173 PMC5738272

[43] Kumar P Lakshmi YS Kondapi AK. Triple drug combination of zidovudine, efavirenz and lamivudine loaded lactoferrin nanoparticles: an effective nano first-line regimen for HIV therapy. Pharm Res. (2017) 34:257–68. 10.1007/s11095-016-2048-4 27928647

[44] Cao S Slack SD Levy CN Hughes SM Jiang Y Yogodzinski C. Hybrid nanocarriers incorporating mechanistically distinct drugs for lymphatic CD4^+^ T cell activation and HIV-1 latency reversal. Sci Adv. (2019) 5:eaav6322. 10.1126/sciadv.aav6322 30944862 PMC6436934

[45] Battivelli E Dahabieh MS Abdel-Mohsen M Svensson JP Da Silva IT Cohn LB. Distinct chromatin functional states correlate with HIV latency reactivation in infected primary CD4^+^ T cells. Elife. (2018) 7:e34655. 10.7554/eLife.34655 29714165 PMC5973828

[46] Jayant RD Atluri VS Agudelo M Sagar V Kaushik A Nair M. Sustained-release nanoART formulation for the treatment of neuroAIDS. Int J Nanomed. (2015) 10:1077–93. 10.2147/IJN.S76517 25709433 PMC4327567

[47] Tang X Liang Y Liu X Zhou S Liu L Zhang F. PLGA-PEG nanoparticles coated with anti-CD45RO and loaded with HDAC plus protease inhibitors activate latent HIV and inhibit viral spread. Nanoscale Res Lett. (2015) 10:413. 10.1186/s11671-015-1112-z 26489856 PMC4614850

[48] Rostami H Ebtekar M Ardestani MS Yazdi MH Mahdavi M. Co-utilization of a TLR5 agonist and nano-formulation of HIV-1 vaccine candidate leads to increased vaccine immunogenicity and decreased immunogenic dose: a preliminary study. Immunol Lett. (2017) 187:19–26. 10.1016/j.imlet.2017.05.002 28479111

[49] Tokatlian T Read BJ Jones CA Kulp DW Menis S Chang JYH. Innate immune recognition of glycans targets HIV nanoparticle immunogens to germinal centers. Science. (2019) 363:649–54. 10.1126/science.aat9120 30573546 PMC6420719

[50] Lori F Calarota SA Lisziewicz J. Nanochemistry-based immunotherapy for HIV-1. Curr Med Chem. (2007) 14:1911–9. 10.2174/092986707781368513 17691933

[51] Jardine JG Kulp DW Havenar-Daughton C Sarkar A Briney B Sok D. HIV-1 broadly neutralizing antibody precursor B cells revealed by germline-targeting immunogen. Science. (2016) 351:1458–63. 10.1126/science.aad9195 27013733 PMC4872700

[52] Martinez-Murillo P Tran K Guenaga J Lindgren G Adori M Feng Y. Particulate array of well-ordered HIV clade C env trimers elicits neutralizing antibodies that display a unique V2 cap approach. Immunity. (2017) 46:804–17.e7. 10.1016/j.immuni.2017.04.021 28514687 PMC5528178

[53] Sliepen K Han BW Bontjer I Mooij P Garces F Behrens AJ. Structure and immunogenicity of a stabilized HIV-1 envelope trimer based on a group-M consensus sequence. Nat Commun. (2019) 10:2355. 10.1038/s41467-019-10262-5 31142746 PMC6541627

[54] Brouwer PJM Antanasijevic A Berndsen Z Yasmeen A Fiala B Bijl TPL. Enhancing and shaping the immunogenicity of native-like HIV-1 envelope trimers with a two-component protein nanoparticle. Nat Commun. (2019) 10:4272. 10.1038/s41467-019-12080-1 31537780 PMC6753213

[55] Dubrovskaya V Tran K Ozorowski G Guenaga J Wilson R Bale S. Vaccination with glycan-modified HIV NFL envelope trimer-liposomes elicits broadly neutralizing antibodies to multiple sites of vulnerability. Immunity. (2019) 51:915–29.e7. 10.1016/j.immuni.2019.10.008 31732167 PMC6891888

[56] He L de Val N Morris CD Vora N Thinnes TC Kong L. Presenting native-like trimeric HIV-1 antigens with self-assembling nanoparticles. Nat Commun. (2016) 7:12041. 10.1038/ncomms12041 27349934 PMC4931238

[57] Lara HH Ayala-Nunez NV Ixtepan-Turrent L Rodriguez-Padilla C. Mode of antiviral action of silver nanoparticles against HIV-1. J Nanobiotechnology. (2010) 8:1. 10.1186/1477-3155-8-1 20145735 PMC2818642

[58] Sheik DA Chamberlain JM Brooks L Clark M Kim YH Leriche G. Hydrophobic nanoparticles reduce the β-sheet content of SEVI amyloid fibrils and inhibit SEVI-enhanced HIV infectivity. Langmuir. (2017) 33:2596–602. 10.1021/acs.langmuir.6b04295 28207276

[59] Wei X Zhang G Ran D Krishnan N Fang RH Gao W. T-cell-mimicking nanoparticles can neutralize HIV infectivity. Adv Mater. (2018) 30:e1802233. 10.1002/adma.201802233 30252965 PMC6334303

[60] Nahui Palomino RA Vanpouille C Laghi L Parolin C Melikov K Backlund P. Extracellular vesicles from symbiotic vaginal lactobacilli inhibit HIV-1 infection of human tissues. Nat Commun. (2019) 10:5656. 10.1038/s41467-019-13468-9 31827089 PMC6906448

[61] Welch JL Kaddour H Winchester L Fletcher CV Stapleton JT Okeoma CM. Semen extracellular vesicles from HIV-1-infected individuals inhibit HIV-1 replication *in vitro*, and extracellular vesicles carry antiretroviral drugs *in vivo*. J Acquir Immune Defic Syndr. (2020) 83:90–8. 10.1097/QAI.0000000000002233 31809364 PMC7144830

[62] Shahbazi R Sghia-Hughes G Reid JL Kubek S Haworth KG Humbert O. Targeted homology-directed repair in blood stem and progenitor cells with CRISPR nanoformulations. Nat Mater. (2019) 18:1124–32. 10.1038/s41563-019-0385-5 31133730 PMC6754292

[63] Dash PK Kaminski R Bella R Su H Mathews S Ahooyi TM. Sequential LASER ART and CRISPR treatments eliminate HIV-1 in a subset of infected humanized mice. Nat Commun. (2019) 10:2753. 10.1038/s41467-019-10366-y 31266936 PMC6606613

[64] Sweeney EEBP Balakrishnan PB Powell AB Bowen A Sarabia I Burga RA PLGA nanodepots co-encapsulating prostratin and anti-CD25 enhance primary natural killer cell antiviral and antitumor function. Nano Res. (2020) 13:736–44. 10.1007/s12274-020-2684-1 PMC8168447 34079616

[65] Jones RB Mueller S Kumari S Vrbanac V Genel S Tager AM. Antigen recognition-triggered drug delivery mediated by nanocapsule-functionalized cytotoxic T-cells. Biomaterials. (2017) 117:44–53. 10.1016/j.biomaterials.2016.11.048 27936416 PMC5204257

[66] Aroh C Wang ZH Dobbs N Luo M Chen ZJ Gao JM. Innate immune activation by cGMP-AMP nanoparticles leads to potent and long-acting antiretroviral response against HIV-1. J Immunol. (2017) 199:3840–8. 10.4049/jimmunol.1700972 29084836 PMC5916791

[67] Iannazzo D Pistone A Ferro S De Luca L Monforte AM Romeo R. Graphene quantum dots based systems as HIV inhibitors. Bioconjugate Chem. (2018) 29:3084–93. 10.1021/acs.bioconjchem.8b00448 30106563

[68] Roy U Drozd V Durygin A Rodriguez J Barber P Atluri V. Characterization of nanodiamond-based anti-HIV drug delivery to the brain. Sci Rep. (2018) 8:1063. 10.1038/s41598-017-16703-9 29371638 PMC5785470

[69] Garrido C Simpson CA Dahl NP Bresee J Whitehead DC Lindsey EA. Gold nanoparticles to improve HIV drug delivery. Future Med Chem. (2015) 7:1097–107. 10.4155/fmc.15.57 26132521 PMC4501014

[70] Mandal S Belshan M Holec A Zhou Y Destache CJ. An enhanced emtricitabine-loaded long-acting nanoformulation for prevention or treatment of HIV infection. Antimicrob Agents Chemother. (2017) 61:e01475-16. 10.1128/AAC.01475-16 27821449 PMC5192106

[71] Destache CJ Mandal S Yuan Z Kang GB Date AA Lu WX. Topical tenofovir disoproxil fumarate nanoparticles prevent HIV-1 vaginal transmission in a humanized mouse model. Antimicrob Agents Chemother. (2016) 60:3633–9. 10.1128/AAC.00450-16 27044548 PMC4879396

[72] Mandal S Khandalavala K Pham R Bruck P Varghese M Kochvar A. Cellulose acetate phthalate and antiretroviral nanoparticle fabrications for HIV pre-exposure prophylaxis. Polymers-Basel. (2017) 9:423–40. 10.3390/polym9090423 30450244 PMC6239201

[73] Deeks SG. HIV: shock and kill. Nature. (2012) 487:439–40. 10.1038/487439a 22836995

[74] Spivak AM Planelles V. Novel latency reversal agents for HIV-1 cure. Annu Rev Med. (2018) 69:421–36. 10.1146/annurev-med-052716-031710 29099677 PMC5892446

[75] Qatsha KA Rudolph C Marme D Schachtele C May WS. Go-6976, a selective inhibitor of protein-kinase-C, is a potent antagonist of human immunodeficiency virus-1 induction from latent low-level-producing reservoir cells-*in vitro*. Proc Natl Acad Sci USA. (1993) 90:4674–8. 10.1073/pnas.90.10.4674 7685108 PMC46575

[76] Kovochich M Marsden MD Zack JA. Activation of latent HIV using drug-loaded nanoparticles. PLoS ONE. (2011) 6:e18270. 10.1371/journal.pone.0018270 21483687 PMC3071729

[77] Buchbinder SP Mehrotra DV Duerr A Fitzgerald DW Mogg R Li D. Efficacy assessment of a cell-mediated immunity HIV-1 vaccine (the step study): a double-blind, randomised, placebo-controlled, test-of-concept trial. Lancet. (2008) 372:1881–93. 10.1016/S0140-6736(08)61591-3 19012954 PMC2721012

[78] Haynes BF Gilbert PB McElrath MJ Zolla-Pazner S Tomaras GD Alam SM. Immune-correlates analysis of an HIV-1 vaccine efficacy trial. N Engl J Med. (2012) 366:1275–86. 22475592 10.1056/NEJMoa1113425 PMC3371689

[79] Ada GL. The ideal vaccine. World J Microb Biot. (1991) 7:105–9. 10.1007/BF00328978 24424920

[80] Zaman M Good MF Toth I. Nanovaccines and their mode of action. Methods. (2013) 60:226–31. 10.1016/j.ymeth.2013.04.014 23623821

[81] Lopez-Sagaseta J Malito E Rappuoli R Bottomley MJ. Self-assembling protein nanoparticles in the design of vaccines. Comput Struct Biotec. (2016) 14:58–68. 10.1016/j.csbj.2015.11.001 26862374 PMC4706605

[82] Huleatt JW Jacobs AR Tang J Desai P Kopp EB Huang Y. Vaccination with recombinant fusion proteins incorporating toll-like receptor ligands induces rapid cellular and humoral immunity. Vaccine. (2007) 25:763–75. 10.1016/j.vaccine.2006.08.013 16968658

[83] Rodriguez B Asmuth DM Matining RM Spritzler J Jacobson JM Mailliard RB. Safety, tolerability, and immunogenicity of repeated doses of dermavir, a candidate therapeutic HIV vaccine, in HIV-infected patients receiving combination antiretroviral therapy: results of the ACTG 5176 trial. J Acquir Immune Defic Syndr. (2013) 64:351–9. 10.1097/QAI.0b013e3182a99590 24169120 PMC3858388

[84] Ren RX Yin SW Lai BL Ma LZ Wen JY Zhang XX. Myricetin antagonizes semen-derived enhancer of viral infection (SEVI) formation and influences its infection-enhancing activity. Retrovirology. (2018) 15:49. 10.1186/s12977-018-0432-3 30012153 PMC6048764

[85] Roan NR Munch J Arhel N Mothes W Neidleman J Kobayashi A. The cationic properties of SEVI underlie its ability to enhance human immunodeficiency virus infection. J Virol. (2009) 83:73–80. 10.1128/JVI.01366-08 18945786 PMC2612336

[86] Capule CC Yang J. Enzyme-linked immunosorbent assay-based method to quantify the association of small molecules with aggregated amyloid peptides. Anal Chem. (2012) 84:1786–91. 10.1021/ac2030859 22243436 PMC3277653

[87] Giacomelli CE Norde W. Conformational changes of the amyloid β-peptide (1-40) adsorbed on solid surfaces. Macromol Biosci. (2005) 5:401–7. 10.1002/mabi.200400189 15889393

[88] Moores B Drolle E Attwood SJ Simons J Leonenko Z. Effect of surfaces on amyloid fibril formation. PLoS ONE. (2011) 6:e25954. 10.1371/journal.pone.0025954 22016789 PMC3189948

[89] Hu CM Zhang L Aryal S Cheung C Fang RH Zhang L. Erythrocyte membrane-camouflaged polymeric nanoparticles as a biomimetic delivery platform. Proc Natl Acad Sci USA. (2011) 108:10980–5. 10.1073/pnas.1106634108 21690347 PMC3131364

[90] Campbell EM Hope TJ. HIV-1 capsid: the multifaceted key player in HIV-1 infection. Nat Rev Microbiol. (2015) 13:471–83. 10.1038/nrmicro3503 26179359 PMC4876022

[91] Yanez-Mo M Siljander PR Andreu Z Zavec AB Borras FE Buzas EI. Biological properties of extracellular vesicles and their physiological functions. J Extracell Vesicles. (2015) 4:27066. 10.3402/jev.v4.27066 25979354 PMC4433489

[92] Ma Y Zhang L Huang X. Genome modification by CRISPR/Cas9. FEBS J. (2014) 281:5186–93. 10.1111/febs.13110 25315507

[93] Lino CA Harper JC Carney JP Timlin JA. Delivering CRISPR: a review of the challenges and approaches. Drug Deliv. (2018) 25:1234–57. 10.1080/10717544.2018.1474964 29801422 PMC6058482

[94] Li C Guan X Du T Jin W Wu B Liu Y. Inhibition of HIV-1 infection of primary CD4^+^ T-cells by gene editing of CCR5 using adenovirus-delivered CRISPR/Cas9. J Gen Virol. (2015) 96:2381–93. 10.1099/vir.0.000139 25854553

[95] Xu L Yang H Gao Y Chen Z Xie L Liu Y. CRISPR/Cas9-mediated CCR5 ablation in human hematopoietic stem/progenitor cells confers HIV-1 resistance *in vivo*. Mol Ther. (2017) 25:1782–9. 10.1016/j.ymthe.2017.04.027 28527722 PMC5542791

[96] Lee B Lee K Panda S Gonzales-Rojas R Chong A Bugay V. Nanoparticle delivery of CRISPR into the brain rescues a mouse model of fragile X syndrome from exaggerated repetitive behaviours. Nat Biomed Eng. (2018) 2:497–507. 10.1038/s41551-018-0252-8 30948824 PMC6544395

[97] Lopalco L. CCR5: from natural resistance to a new anti-HIV strategy. Viruses. (2010) 2:574–600. 10.3390/v2020574 21994649 PMC3185609

[98] Lam S Bollard C. T-cell therapies for HIV. Immunotherapy. (2013) 5:407–14. 10.2217/imt.13.23 23557423 PMC3697835

[99] Patel S Jones RB Nixon DF Bollard CM. T-cell therapies for HIV: preclinical successes and current clinical strategies. Cytotherapy. (2016) 18:931–42. 10.1016/j.jcyt.2016.04.007 27265874 PMC4935558

[100] Sung JA Patel S Clohosey ML Roesch L Tripic T Kuruc JD. HIV-Specific, ex vivo expanded T cell therapy: feasibility, safety, and efficacy in ART-suppressed HIV-infected individuals. Mol Ther. (2018) 26:2496–506. 10.1016/j.ymthe.2018.08.015 30249388 PMC6171327

[101] Yan N Regalado-Magdos AD Stiggelbout B Lee-Kirsch MA Lieberman J. The cytosolic exonuclease TREX1 inhibits the innate immune response to human immunodeficiency virus type 1. Nat Immunol. (2010) 11:1005–U53. 10.1038/ni.1941 20871604 PMC2958248

[102] Gao DX Wu JX Wu YT Du FH Aroh C Yan N. Cyclic GMP-AMP synthase is an innate immune sensor of HIV and other retroviruses. Science. (2013) 341:903–6. 10.1126/science.1240933 23929945 PMC3860819

[103] Mansur HS. Quantum dots and nanocomposites. Wires Nanomed Nanobi. (2010) 2:113–29. 10.1002/wnan.78 20104596

[104] Matea CT Mocan T Tabaran F Pop T Mosteanu O Puia C. Quantum dots in imaging, drug delivery and sensor applications. Int J Nanomed. (2017) 12:5421–31. 10.2147/IJN.S138624 28814860 PMC5546783

[105] Garrido C Margolis DM. Translational challenges in targeting latent HIV infection and the CNS reservoir problem. J Neurovirol. (2015) 21:222–6. 10.1007/s13365-014-0269-z 25060298 PMC4305502

[106] Anderson PL Glidden DV Liu A Buchbinder S Lama JR Guanira JV. Emtricitabine-tenofovir concentrations and pre-exposure prophylaxis efficacy in men who have sex with men. Sci Trans Med. (2012) 4:151ra125. 10.1126/scitranslmed.3004006 22972843 PMC3721979

[107] Valade E Treluyer JM Bouazza N Ghosn J Foissac F Benaboud S. Population pharmacokinetics of emtricitabine in HIV-1-infected adult patients. Antimicrob Agents Chemother. (2014) 58:2256–61. 10.1128/AAC.02058-13 24492366 PMC4023733

